# High-throughput metabolomics exploring the pharmacological effects and mechanism of icariin on rheumatoid arthritis rat based on ultrahigh-performance liquid chromatography coupled with quadrupole time-of-flight mass spectrometry

**DOI:** 10.3389/fmolb.2025.1514882

**Published:** 2025-04-09

**Authors:** Peng Zhou, Xixi Liu, Yushi Tian, Shouze Ren, Hua Liang

**Affiliations:** ^1^ School of Continuing Education, Heilongjiang University of Chinese Medicine, Harbin, China; ^2^ Beijing Mentougou District of Traditional Chinese Medicine, Beijing, China; ^3^ Department of Chinese Formulae, Heilongjiang University of Chinese Medicine, Harbin, China

**Keywords:** metabolomics, multivariate analysis, metabolites, pathways, liquid chromatography, mass spectrometry

## Abstract

**Introduction:**

Metabolomics could provide insights into the pharmacological effects and action mechanisms of drugs through assessment of the changes in relevant biomarkers and biological pathways. Icariin (ICA) is a promising ffavonoid compound known to have significant anticancer activity; however, the pharmacological mechanisms of ICA in the treatment of rheumatoid arthritis (RA) need to be explored further.

**Methods:**

The changes in the metabolic profiles of serum samples were revealed using non-targeted metabolomics based on ultrahigh-performance liquid chromatography coupled with quadrupole time-of-fight mass spectrometry. Tissue histopathology, physical parameters, and biochemical indicators were also measured and analyzed to reveal the mechanisms of ICA in the treatment of RA.

**Results and discussion:**

Thirty-one potential biomarkers were identified to highlight the metabolic disorders in an RA animal model, out of which twenty-three were regulated by ICA treatment. These biomarkers were mainly involved in alanine, aspartate, and glutamate metabolism; arachidonic acid metabolism; citrate cycle; pyruvate metabolism; and glycolysis/gluconeogenesis pathways. The anticancer mechanism of ICA on RA may be attributed to amelioration of the amino acid metabolism, unsaturated fatty acid metabolism, citrate cycle, pyruvate metabolism, and others, which in turn regulate the oxidative stress state and inflammatory effects. Thus, metabolomics is a promising approach for revealing the biomarker distribution and pathways of RA to determine the effects and mechanisms of ICA, which can benefit the development of natural medicines.

## 1 Introduction

Rheumatoid arthritis (RA) is a chronic autoimmune disease caused by immune dysregulation in the body, leading to synovial inflammation and bone erosion. Beyond the clinical manifestations in the bones and joints, RA can also affect multiple systems throughout the body, resulting in various complications (Díaz-González and Hernández-Hernández, 2023; Smith and Berman, 2022). The World Health Organization (WHO) has confirmed that RA is the 31st leading cause of years lived with disability (YLD) globally (Monti et al., 2015). The global incidence of RA ranges from 0.5% to 1%. In China, almost 82% of RA patients exhibit moderate to high disease activities, and about 43.48% of patients among those who have had the disease for 5–10 years suffer from functional impairments and joint deformities; this proportion increases to 61.25% for those patients who have suffered from the disease for over 15 years, imposing significant negative impacts on both the patients and society (Tian et al., 2021). The pathogenesis of RA is demonstrated to be intimately associated with genetic and environmental factors, cytokines, inflammatory signaling pathways, immune cells, and intestinal dysbiosis (Venetsanopoulou et al., 2022; Zhao et al., 2022; Jang et al., 2022; Seymour et al., 2024). In typical RA, the pathological changes in the synovium involve formation of pannus and infiltration of the immune cells, which subsequently lead to the production of matrix metalloproteinases that degrade the surrounding cartilage and bone (Zhang et al., 2023; Gan et al., 2024). This process imposes high demands on energy and biosynthetic precursors, suggesting that the metabolic alterations constitute a fundamental disease mechanism of RA (Xu et al., 2022). Currently, the clinical treatment of RA mainly entails pharmacotherapy (Nasonov, 2016); in particular, non-steroidal anti-inflammatory drugs (NSAIDs) serve as the first-line treatment for RA to minimize joint pain, stiffness, and inflammation by downregulating the pro-inflammatory cytokines, such as prostaglandins and thromboxane. However, these drugs fail to address the underlying causes and may elicit gastrointestinal disturbances, asthma, and nephrotoxicity (Bindu et al., 2020; LaForge et al., 2023; GBD, 2021 Rheumatoid Arthritis Collaborators, 2023). Glucocorticoids and disease-modifying anti-rheumatic drugs (DMARDs) are known to effectively alleviate joint swelling, pain, and systemic symptoms in RA patients, albeit with notable limitations in terms of the duration of use and safety (Yap et al., 2018; Zago et al., 2020; Doumen et al., 2023).

It is an urgent requirement in the field of autoimmune disease research to develop better anti-RA agents as new treatment strategies to enhance disease prognosis and selectively aim at the targets and specific pathways of RA treatment. Medicinal plants and herbs have attracted increasing interest from the scientific community in the hope that emerging chemical ingredients that can address multiple targets at low cost, are easy to obtain, are relatively free of toxicity, and have high usability will be found to mitigate the limitations of the currently available drugs (Talari et al., 2014; Hussein and Khalifa, 2014; Mnayer et al., 2014). Icariin (ICA) is a bioactive monomer belonging to flavonoid glycosides extracted from the Epimedium species of plants (Tan et al., 2016). Numerous studies have shown that ICA is involved in anti-inflammatory and immune responses in some diseases, such as multiple sclerosis, asthma, atherosclerosis, lupus nephritis, inflammatory bowel diseases, and cancer (Shen and Wang, 2018; Bi et al., 2022). In RA, ICA has been observed to inhibit the migration and proliferation of fibroblast-like synoviocytes (FLSs) in a concentration-dependent manner by inducing G2/M phase arrest and apoptosis; additionally, it also reduces the mitochondrial transmembrane potential, upregulates cytosolic cytochrome c, and increases the level of intracellular reactive oxygen species (Pu et al., 2021). Several studies have shown that ICA inhibits cell proliferation and inflammatory responses while promoting the apoptosis of RA-FLS cells by regulating the TRIB1/TLR2/NF-kB pathway, GAREM1/MAPK pathway, miR-223-3p/NLRP3 pathway, and others (Wu et al., 2022; Wu et al., 2024; Wu et al., 2020). However, the mechanism of ICA in the treatment of RA is not yet fully elucidated.

**FIGURE 1:**
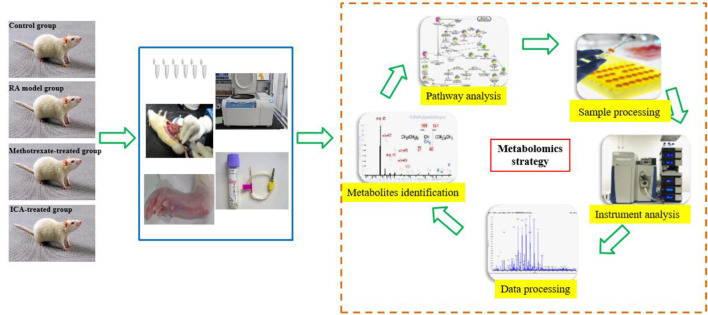
Schematic illustration of the animal experimental design using high-throughput metabolomics.

Metabolomics is a rapidly emerging field of omics after the age of genomics and proteomics; it is concerned with the qualitative and quantitative analyses of small endogenous metabolites from biofluids, tissues, and cells with molecular weights less than 1,500 Da to provide integral information regarding the associated biomarkers and pathways (Rinschen et al., 2019; Lanznaster et al., 2018). Metabolites are the final downstream products of metabolism and possess outstanding abilities to depict the occurrence and development of illness compared to other omics approaches (Dickie et al., 2017; Yang et al., 2018a). Non-targeted metabolomics has been used for all-sided reportage analysis of metabolomes under suppose–generating circumstances (Mu et al., 2019; Sun et al., 2018a; Fang et al., 2019; Sun et al., 2018b; Qiu et al., 2025; Xie et al., 2019; Wang et al., 2025). Targeted metabolomics involves quantitative measurements of specific metabolites, such as saccharides, fatty acids, lipids, and amino acids, which are typically used to detect the metabolic pathways of a disease or evaluate distinguished metabolites obtained through non-targeted metabolic research (Varma et al., 2018; Ribbenstedt et al., 2018). Generally, metabolomics research relies on various high-throughput techniques (Mo et al., 2020; French et al., 2018; Dai et al., 2020). Ultrahigh-performance liquid chromatography coupled with quadrupole time-of-flight mass spectrometry (UHPLC-QTOF-MS) has been widely used in metabolomics because of its high sensitivity and selectivity, better peak resolution, and low ion suppression, which have allowed determination of accurate chemical information on thousands of compounds related to responses to endogenous and exogenous impetuses, such as food, drugs, and pathogens (Sun et al., 2019). Given the time-consuming, laborious, and difficult nature of recruiting patients to detect the action mechanisms of drugs and pro-drugs in clinical practice, a rat model is considered easier and more repeatable for conducting relevant experiments; accordingly, it is common practice to first compare the similarities between human patients and the rat model to obtain specific potential biomarkers and metabolic pathways before conducting further clinical research (Hu et al., 2017). To explore the pharmacological effects and mechanisms of ICA against the RA rat, serum metabolomics was conducted in this study using UHPLC-QTOF-MS at high resolution to assess the changes in the relevant biomarkers and biological pathways (Figure 1).

## 2 Materials and methods

### 2.1 Materials and reagents

Complete Freund’s adjuvant (CFA) was purchased from Beijing Solarbio Technology Co., Ltd., (Beijing, China). Icariin (purity >98.9%) was obtained from Northern Biotechnology Research Institute (Beijing, China). The ELISA kits for interleukin-6 (IL-6), tumor necrosis factor-α (TNF-α), and matrix metallopeptidase-3 (MMP-3) were obtained from Omega Bio-Tek, Inc. (Norcross, GA, United States). The ELISA kits for alanine aminotransferase (ALT), aspartate aminotransferase (AST), catalase (CAT), superoxide dismutase (SOD), and malondialdehyde (MDA) were purchased from Biodiagnostics (Cairo, Egypt). The ELISA kits for lactate dehydrogenase (LDH), glutathione-S-transferase (GST), and glutathione peroxidase (GSH-PX) were purchased from the Institute of Bioengineering (Nanjing, China). Pentobarbital sodium, 10% neutral buffered formalin, and sodium chloride injection were purchased from Shanghai Chemical Reagent Purchasing and Supply Station, China.

Chromatographic-grade pure acetonitrile and methanol were obtained from Merck (Darmstadt, Germany); in addition, chromatographic-grade pure formic acid and leucine enkephalin were purchased from Invitrogen Life Technologies (Carlsbad, CA, United States). Water without ions was obtained using a Milli-Q ultrapure water system (Millipore, Bedford, MA, United States). All other reagents and chemicals used were of analytical grade and were acquired from East Biotechnology Research Institute (Beijing, China) or Sanlian Pharmaceutical Co., Ltd., (Harbin, China).

### 2.2 RA animal model and treatment

Forty clean 4-week-old SD male rats weighing 140 ± 20 g were obtained from the Experimental Animal Center of Beijing University of Traditional Chinese Medicine. They were then raised for 7 d in specific pathogen-free cages and exposed to 12-h light/dark cycles from 08:00 to 20:00 at controlled temperatures of 24°C ± 2°C and humidity of 50% ± 5% for acclimatization with free access to water and standard feed. The SD rats were randomly divided into four groups (10 rats per group) as the control, RA model, methotrexate-treated, and ICA-treated groups. CFA was retrieved from 4°C refrigeration and shaken for 5 min. The rats were then injected with 0.1 mL of CFA at a concentration of 2.0 mg/mL once in the right hind foot, except for the control group, and the best injection was noted to produce white bubbles locally. Then, 0.1 mL of physiological saline was injected in the same manner to the animals in the control group (Zhou et al., 2022; Chen et al., 2024). After 14 days of modeling, the ICA-treated and methotrexate-treated groups were, respectively, given 8 mg·kg^−1^ and 0.4 mg·kg^−1^suspension once a day in the morning for 2 weeks. Correspondingly, the animals in the control group were administered sodium chloride solution orally.

### 2.3 Sampling and processing

Twenty-four hours after the final successive drug treatment, the animals in all groups were subjected to mild ether anesthesia, and blood samples were collected from the aorta abdominalis. Subsequently, the blood samples were allowed to coagulate for 15 min and centrifuged at 4,200 rpm for 10 min at 4°C to separate the serum. The extracted serum was immediately stored at −80°C for metabolomics analysis and routine biochemical index detection. For the metabolomics analysis, the serum samples needed to be processed further, so they were thawed at 4°C before use and diluted with ice-cold methanol in the ratio of 1:4 to precipitate the protein; the mixture was then blended for 30 s using a mixer mill (Retsch GmbH and Co, Haan, Germany) and centrifuged at 4,200 rpm for 15 min at 4°C. The obtained supernatant was transferred into new centrifuge tubes and evaporated to dryness under nitrogen at 22°C ± 3°C before being redissolved in 200 μL of water. Before the UHPLC-QTOF-MS analysis, the samples to be measured were centrifuged at 4,200 rpm for 15 min at 4°C and passed through a 0.22-μm polytetrafluoroethylene filter. Next, 15 μL of each rat serum sample was added to 3,000 μL of the working solution to obtain a quality control (QC) sample for ratifying and optimizing the chromatographic and mass spectrometry (MS) conditions. After blood collection, the liver tissues of all groups of animals were removed, washed with ice-cold phosphate-buffered saline (PBS) to eliminate any extraneous matter, blotted dry, and cut into small sections for biochemical index detection.

### 2.4 Parameter measurements

The serum parameters CAT, LDH, GST, AST, ALT, IL-6, TNF-α, and MMP-3 were measured using the corresponding kits according to the manufacturer's instructions. The GSH-PX content and SOD activity in the liver were estimated according to the method in a reference work, and the level of lipid peroxidation in the liver tissue was measured as the MDA activity (Elguindy et al., 2016).

### 2.5 Metabolomics experiment

#### 2.5.1 Liquid chromatography conditions

The serum samples from the different groups were interpreted using an ACQUITY H-CLASS instrument (Waters Corporation, Milford, MA, United States) comprising a quaternary pump, an automatic degasser, and an autosampler before being passed to an ACQUITY UPLC™ HSS T3 column (100 × 2.1 mm, 1.8 μm; Waters Corporation) for chromatographic separation at a flow rate of 0.3 mL/min. The injection volume was controlled to 4 μL, and the column oven was maintained at 35°C. The best mobile system included 0.1% formic acid/water (A) and 0.1% formic acid/acetonitrile (B), and the mobile phase gradient was set as follows: 0–1 min, 2%–15% B; 1–3 min, 15%–45% B; 3–7 min, 45%–70% B; 7–9 min, 70%–90% B; 9–10 min, 90%–95% B; 10–12 min 95%–2%; 12–14 min 2%B. The QC sample was injected six times at the beginning of the run in the UHPLC-QTOF-MS operation, and each of the eight samples were detected again to ensure data endurance and persistence. Nitrogen was sprayed at 50 L/h and also applied as an eluent gas at 550 L/h. The exported liquid was directly delivered to the MS section without separation.

#### 2.5.2 Mass spectrometry conditions

MS detection was performed on a Triple TOF™ 5600+ system (equipped with a DuoSpray Ion source) matched with ion electrospray sources in both positive and negative modes (AB SCIEX, Foster City, CA, United States). The main parameters in the positive mode were set as follows: ion spray voltage, 5 kV; ion source temperature, 400°C; curtain gas, 30 psi; nebulizer gas (GS 1) and heater gas (GS 2), 50 psi; declustering potential (DP), 70 V; collision energy (CE), 40 eV; collision energy spread (CES), ±15 eV. Meanwhile, the main parameters in the negative mode were set as follows: ion spray voltage, −5 kV; ion source temperature, 400°C; curtain gas, 25 psi; GS 1 and GS 2, 45 psi; DP, −70 V; CE, −30 eV; CES, ±12 eV. Under both ion modes, the mass ranges of the original data were captured in the m/z range of 50–1,500 Da. The positive ion mode ([M+H]+ = 556.2771) and negative ion mode ([M−H]− = 554.2614) were analyzed as LockSpray interference to ensure accuracy of the instrument. Nine characteristic peaks were selected for validation assisted through dynamic background subtraction. For the successive operations of the six replicates of QC samples, the calculated relative standard deviation % (RSD%) values of the retention time (RT) and the peak areas were 0.79% and 2.10%, and these values were 0.96% and 2.43% for the six parallel samples used in the repeatability evaluation, respectively.

#### 2.5.3 Data processing

All serum samples were analyzed by UHPLC-QTOF-MS, and the original data were processed using MassLynx V4.1 software (Waters Corporation, Milford, United States) for peak data extraction, denoising, and selection; isotope and adduct deconvolutions; RT correction; and sample normalization. These data were exported from MassLynx V4.1 software and scaled using a pareto scaling algorithm before being autofitted for multivariate data analysis using SIMCA-P 14.1 (Umetrics, Sweden), including unsupervised principal component analysis (PCA), supervised orthogonal partial least-squares discriminant analysis (OPLS-DA), and variable importance in projection (VIP) score plots. A range of statistical models were utilized to forecast the metabolic phenotypes and distinguish the metabolites. The PCA score plots were used to describe the characteristic that similar metabolomic items display small scatter ranges while heterogeneous metabolomic items are dispersed over large ranges. OPLS-DA was used to highlight the contributions of the captured variables to determine their distinct traits with the most impacts among the different groups; subsequently, we obtained the loading plot and VIP plots to select potential ions. A validation test was performed by calculating the R^2^ and Q^2^ values, which were then used to evaluate the goodness of fit of the partial least-squares models; here, R^2^ close to 1 is the requisite condition for a good model, and Q^2^ exceeding 0.5 represents good predictability of the model. The permutation test was performed to assess the predictability of each model. The ion fragments with small VIP values at the bottom of the VIP scatter plot provide smaller contributions to the differential metabolism between the groups. Conversely, ion fragments with larger VIP values contribute more to the metabolic profiling. In the loading plot, ions that are farther from the original point generated using OPLS-DA are deemed as potential metabolites.

The potential metabolites were deeply screened using two-tailed independent Student's t-tests based on the condition that *p*-value < 0.05 and VIP value >1; the calculations were performed using SPSS Statistics 19.0 (SPSS, Chicago, IL, United States). The metabolites were identified using their RTs; precise MS; tandem MS of standard and online databases such as HMDB, METLIN, Mass Bank, and ChemSpider with PeakView software 1.2.0 and XIC manager. Biological information analysis of the pathways was conducted using MetaboAnalyst 4.0 software and the Kyoto Encyclopedia of Genes and Genomes (KEGG) database. The results of the statistical analyses of different groups using the two-tailed, two-sample Student’s t-test were expressed in terms of the mean and standard deviation (SD) values. Statistical differences <0.05 were regarded as meaningful data, and differences <0.01 were regarded as extremely meaningful data.

## 3 Results

### 3.1 Effects of ICA on RA rat model

**FIGURE 2:**
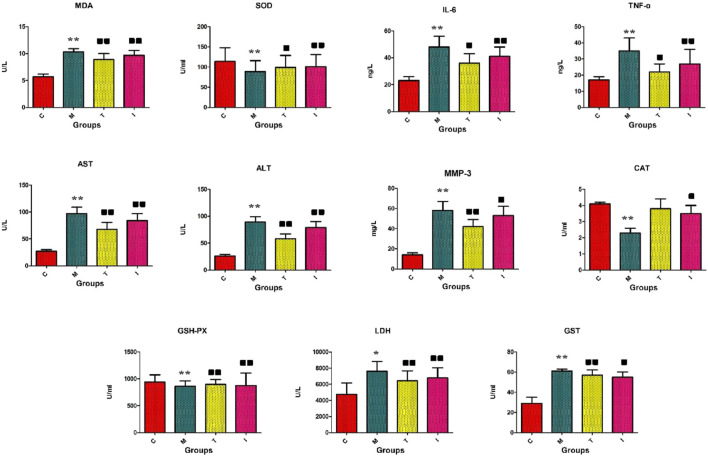
Biochemical indicators analyses of the levels of GSH-PX, SOD, CAT, LDH, GST, MDA, AST, ALT, MMP-3, IL-6, and TNF-α. C, control group; M, rheumatoid arthritis (RA) model group; T, methotrexate-treated group; I, icariin (ICA)-treated group. ∗∗*p* < 0.01 and ∗*p* < 0.05 compared to the control group; 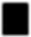 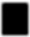 *p* < 0.01 and 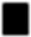 *p* < 0.05 compared to the RA model group.

The rats in the control group were lively with shiny hair, normal eating and drinking trends, naturally increased bodyweights, and granular stools. However, the rats in the model groups showed erect hairs and had less energy, decreased appetite, and lower weight gains. The serum and liver biochemistry results of the model rats are presented in Figures 2. The RA model animals evidently showed morbid states leading to greater concentrations of LDH, GST, AST, ALT, MMP-3, IL-6, TNF-α in the blood, and MDA in the tissues along with decreased concentrations of CAT in the blood as well as GSH-PX and SOD in the tissues compared to the control group. In the inflammatory cells, the reduction of antioxidant levels is always accompanied by increase in reactive oxygen species production. Following intragastric management with ICA, the above abnormal parameters were progressively alleviated, and statistical differences were found in the majority of indexes upon comparison with the RA model group. The serum GSH-PX (*p* < 0.01), SOD (*p* < 0.01), and CAT (*p* < 0.05) concentrations were significantly upregulated, and the levels of LDH (*p* < 0.01), GST (*p* < 0.05), MDA (*p* < 0.01), AST (*p* < 0.01), ALT (*p* < 0.01), MMP-3 (*p* < 0.05), IL-6 (*p* < 0.01), and TNF-α (*p* < 0.01) were significantly downregulated. These results suggest that ICA protects against RA by improving the immune functions, reducing inflammation reactions, and regulating the oxidation–reduction system.

### 3.2 Multivariate statistical analysis of serum metabolomics data

**FIGURE 3:**
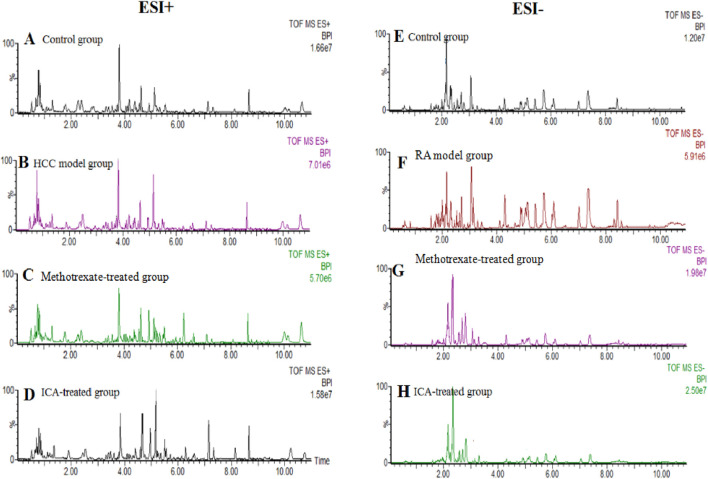
Base peak intensity chromatogram profiles of the **(A, E)** control, **(B, F)** RA model, **(C, G)** methotrexate-treated, and **(D, H)** ICA-treated groups obtained via ultrahigh-performance liquid chromatography coupled with quadrupole time-of-flight mass spectrometry in the positive and negative ion modes, respectively.

**FIGURE 4:**
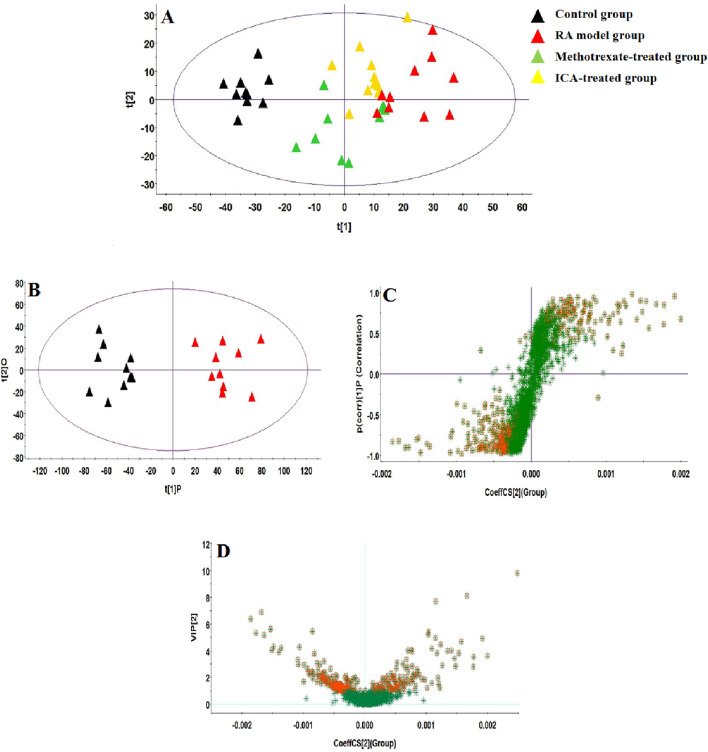
Score plots of the **(A)** principal component analysis (PCA) model of the control, RA, methotrexate-treated, and ICA-treated groups in ESI+ mode; **(B)** orthogonal partial least-squares discriminant analysis (OPLS-DA) of data between the control and RA groups in the ESI+ mode. **(C)** S-plot of the OPLS-DA model for the control and RA groups in ESI+ mode. **(D)** Variable importance in projection (VIP) score plot of the OPLS-DA model for the control and RA groups in ESI+ mode.

**FIGURE 5:**
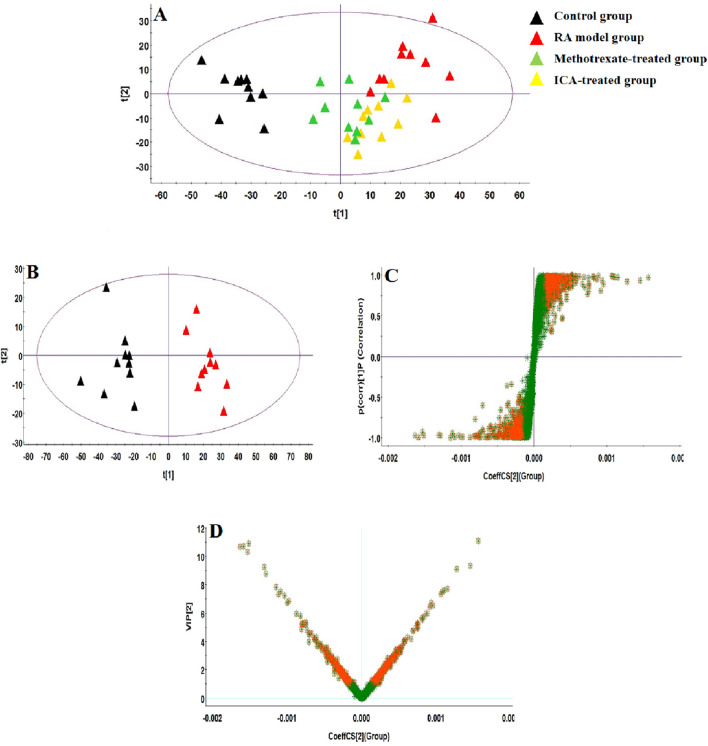
Score plots of the **(A)** PCA model of the control, RA, methotrexate-treated, and ICA-treated groups in ESI– mode; **(B)** OPLS-DA of data between the control and RA groups in ESI– mode. **(C)** S-plot of the OPLS-DA model for the control and RA groups in ESI– mode. **(D)** VIP score plot of the OPLS-DA model for the control and RA groups in ESI– mode.

UHPLC-QTOF-MS analysis offers a rapid, effective, and convenient method of describing the differences in serum metabonomic profiles. Figures 3A–H illustrate the base peak intensity (BPI) chromatograms of the serum samples in the negative and positive ion modes from the control, RA model, methotrexate-treated, and ICA-treated rats, respectively. Intuitively, it is not difficult to observe differences in the chromatographic peak strengths and numbers in the BPI profiles among the control, RA model, and ICA-treated groups, indicating that the metabolic states were hindered by the chemical modeling agent and ICA treatment. Using the UHPLC-QTOF-MS data, PCA was first applied as a common unsupervised statistical method for multivariate data analysis to explore the metabolomic differences between the four groups; this step is characterized by reduced dimensionality and minimal information loss that retains only those features contributing most to the variance. From Figures 4A, 5A, the scattered points of the different samples in the score plots show obvious separation under both negative and positive ion modes, indicating considerable metabolite differences between the four groups. Among these groups, the methotrexate-treated and ICA-treated groups had similar trends and were situated between the trends of the control and RA model groups. To better understand and discern the relationships among the control, RA model, and ICA-treated groups, supervised OPLS-DA was used as a forceful method of selecting the discriminating ions associated with classification of the samples while eliminating the non-correlated variations within the spectra. The score plots shown in Figures 4B, 5B display the results of the OPLS-DA model based on data from the control and RA model groups under the negative and positive ion modes, respectively. Then, the clustering of the RA model group is obviously separable from that of the control group; these indicate that the model is well established for good predictive ability, with R^2^Y (cumulative) of 0.989 and Q^2^ (cumulative) of 0.947 for the ESI+ mode as well as R^2^Y (cumulative) of 0.974 and Q^2^ (cumulative) of 0.938 for the ESI– mode. The S-plots (shown in Figures 4C, 5C) and VIP plots (shown in Figures 4D, 5D) were employed to further determine the contributions of each ion and choose the endogenous metabolites; here, the farther a red symbol is from the origin, the more is its contribution to the group difference.

### 3.3 Identification of potential biomarkers

**FIGURE 6:**
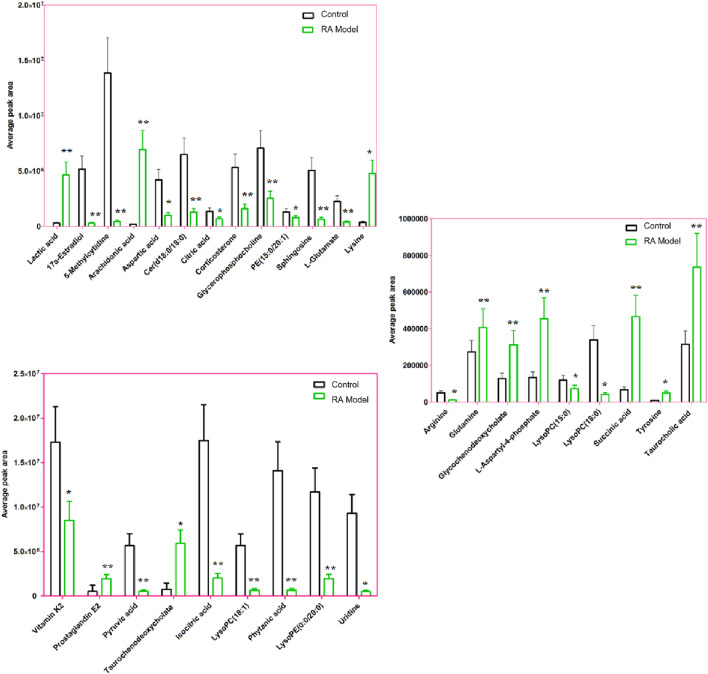
Average peak area changes of 31 potential metabolites in the control and RA model rats. ∗∗*p* < 0.01 and ∗*p* < 0.05 compared to the control group.

The condition of ion selection for metabolite exploration is that the variables have VIP values > 1 during preliminary screening and *p*-values <0.05 in the two-tailed independent Student’s t-test so as to decrease the risk of false positives. A total of 804 variables were selected as the candidates using the threshold of VIP ≥1.0; then, thirty-one candidate biomarkers were obtained in the ESI+ and ESI− mode analyses with significant differences (*p* < 0.05) between the control and RA model groups after filtering. It is worth noting that the criteria were restricted to features with 1.5-fold average normalized intensity differences. The possible molecular formulas and structures of the ions were identified from the online database using the RTs and accurate m/z ratios, which also removes non-endogenous substances. Fragment ions from the targeted MS/MS analysis and corresponding authentic standards were used for structural confirmations. The relevant information regarding the identified biomarkers, including lactic acid, 17a-estradiol, 5-methylcytidine, arachidonic acid, arginine, aspartic acid, cer(d18:0/18:0), citric acid, corticosterone, glutamine, glycerophosphocholine, glycochenodeoxycholate, isocitric acid, L-aspartyl-4-phosphate, L-glutamate, lysine, LysoPC(15:0), LysoPC(18:0), succinic acid, LysoPC(18:1), PE(15:0/20:1), phytanic acid, prostaglandin E2 (PGE2), pyruvic acid, sphingosine, LysoPE(0:0/20:0), taurochenodeoxycholate, taurocholic acid, tyrosine, uridine, and vitamin K2, is recorded in Supplementary Table S1. The average normalized intensity of each biomarker in the control and RA model groups used to obtain the relative intensity information is calculated and shown in Figure 6 for observing the change trends of the discernible biomarkers in RA rats. Compared with the control rats, the serum levels of 11 metabolites, such as lactic acid, arachidonic acid, glutamine, glycochenodeoxycholate, and lysine, were higher while the serum levels of 20 metabolites, such as 17a-estradiol, 5-methylcytidine, arginine, aspartic acid, and citric acid, were lower.

### 3.4 Alterations in potential biomarkers induced by ICA

**FIGURE 7:**
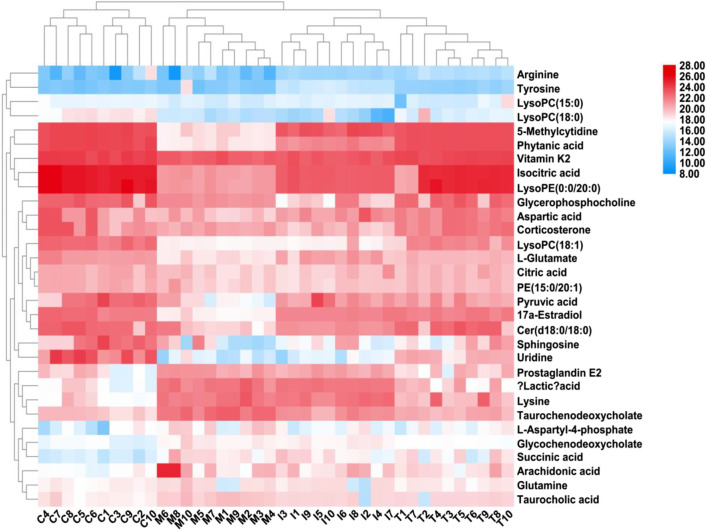
Heatmap describing the trend changes in the control, RA, methotrexate-treated, and ICA-treated groups. The red areas indicate increased content, and the blue areas indicate decreased content; meanwhile, the vertical cluster analysis indicates metabolites with similar chemical properties and types, while the horizontal cluster analysis indicates samples with similar degrees of changes. C, control group; M, RA model group; T, methotrexate-treated group; I, ICA-treated group.

**FIGURE 8:**
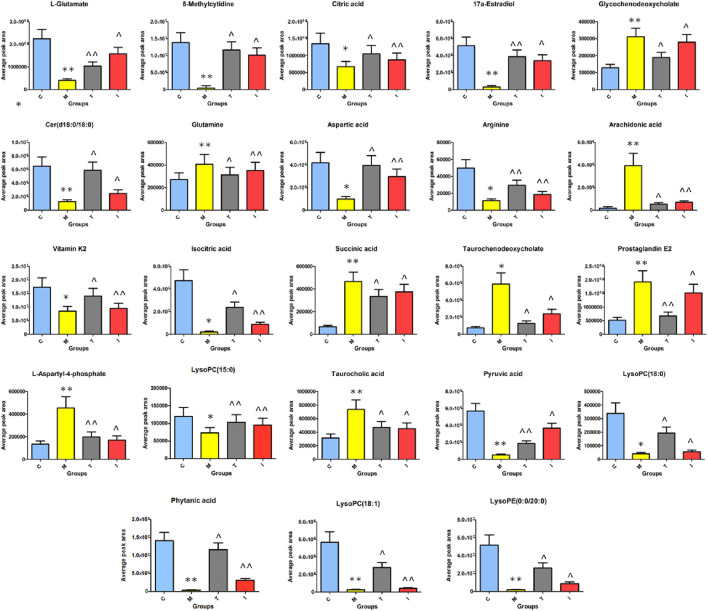
Average peak area changes of 23 potential metabolites in different groups after sixteen successive weeks of ICA treatment. C, control group; M, RA model group; T, methotrexate-treated group; I, ICA-treated group. **p < 0.01 and *p < 0.05 compared to the control group; ^^*p* < 0.01 and ^*p* < 0.05 compared to the RA model group.

After ICA treatment, 23 of the above biomarkers, including 17a-estradiol, 5-methylcytidine, arachidonic acid, arginine, aspartic acid, Cer(d18:0/18:0), citric acid, glutamine, glycochenodeoxycholate, isocitric acid, L-aspartyl-4-phosphate, L-glutamate, LysoPC(15:0), LysoPC(18:0), succinic acid, LysoPC(18:1), phytanic acid, PGE2, pyruvic acid, LysoPE(0:0/20:0), taurochenodeoxycholate, taurocholic acid, and vitamin K2, were found to be altered. A heatmap was established (Figure 7) to describe the changed trends in the four groups, where the dark areas indicate increased content and light areas indicate decreased content; meanwhile, vertical cluster analysis was used to indicate metabolites with similar chemical properties and types, while horizontal cluster analysis was used to indicate samples with similar degrees of changes. As shown in Figure 8, compared with the RA model group, 17a-estradiol, 5-methylcytidine, arginine, aspartic acid, cer(d18:0/18:0), citric acid, isocitric acid, L-glutamate, LysoPC(15:0), LysoPC(18:0), LysoPC(18:1), phytanic acid, pyruvic acid, LysoPE(0:0/20:0), and vitamin K2 levels were significantly upregulated in the ICA-treated group; in contrast, arachidonic acid, glutamine, glycochenodeoxycholate, L-aspartyl-4-phosphate, succinic acid, PGE2, taurochenodeoxycholate, and taurocholic acid levels were significantly downregulated in the ICA-treated group. The changes in the potential biomarkers were similar in the methotrexate-treated and ICA-treated groups. The results show that the concentrations of metabolites in the ICA-treated group were mostly between those in the control and RA model groups, indicating that ICA treatment may assist in protecting against RA by regulating the levels of these metabolites to values close to those in the control group.

### 3.5 Metabolomic pathways and network analysis

**FIGURE 9:**
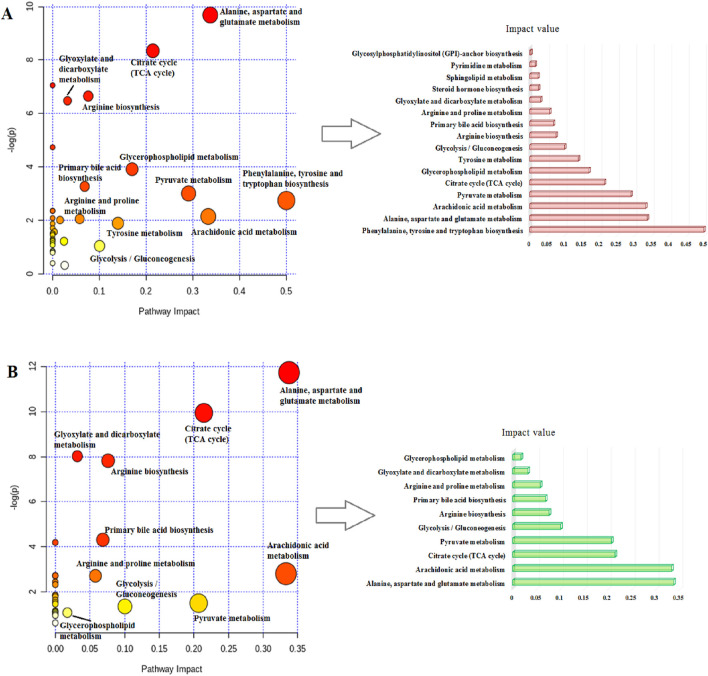
Schematic illustration of the **(A)** disturbed metabolic pathways related to RA and **(B)** after ICA treatment.

**FIGURE 10:**
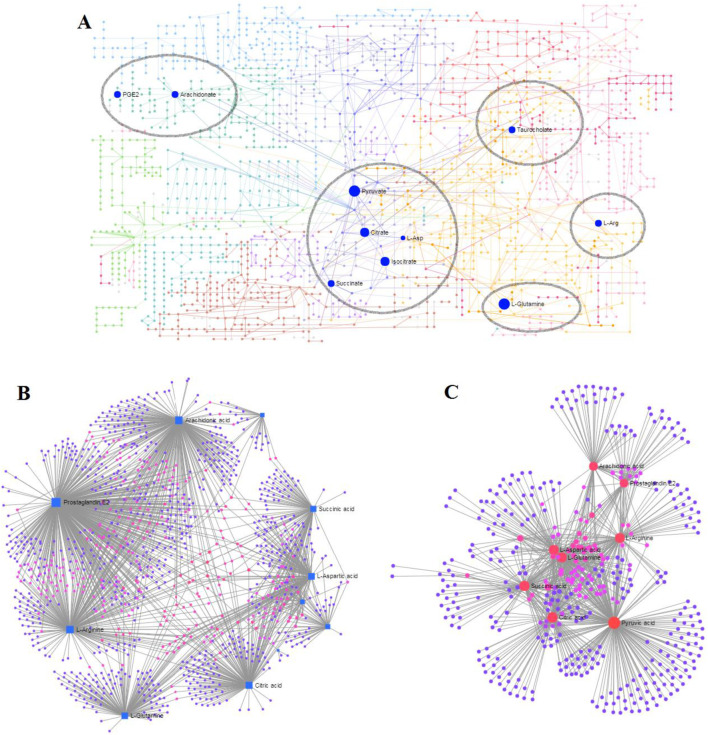
Network analyses associated with ICA efficacy in RA rats: **(A)** KEGG global metabolic network; **(B)** gene–metabolite network; **(C)** metabolite–metabolite network.

MetaboAnalyst as a free web-based tool couples topological and pathway enrichment analyses. Figure 9A displays 31 differential metabolites in the RA model rats with impact values greater than zero that are mainly involved in sixteen pathways. These include phenylalanine, tyrosine, and tryptophan biosynthesis; alanine, aspartate, and glutamate metabolism; arachidonic acid metabolism; pyruvate metabolism; citrate cycle; glycerophospholipid metabolism; tyrosine metabolism; and glycolysis/gluconeogenesis. After ICA treatment, 27 pathways whose metabolite values were mostly greater than zero were generally regulated. These pathways included alanine, aspartate, and glutamate metabolism; arachidonic acid metabolism; citrate cycle; pyruvate metabolism; glycolysis/gluconeogenesis; arginine biosynthesis; primary bile acid biosynthesis; arginine and proline metabolism; glyoxylate and dicarboxylate metabolism; and glycerophospholipid metabolism. The impact values of the above vital metabolic pathways are shown in Figure 9B. KEGG global metabolic network is used to identify the vital mapping metabolites and enzymes/knockouts related to the antitumor effects of ICA, as shown in Figure 10A. These data can be used to integrate metabolomics and metagenomics research and include the alanine, aspartate, and glutamate metabolism; citrate cycle; glyoxylate and dicarboxylate metabolism; primary bile acid biosynthesis; arachidonic acid metabolism; arginine and proline metabolism; D-glutamine and D-glutamate metabolism; and nitrogen metabolism. The gene–metabolites network contains 1,146 nodes and 1,559 edges that mainly comprise 10 metabolites, including PGE2, arachidonic acid, L-arginine, citric acid, L-aspartic acid, L-glutamine, succinic acid, pyruvic acid, isocitric acid, and phytanic acid, and 1,136 genes such as *DECR1*, *PKLR*, *MDH2*, *TAC1*, *LDHA*, *PKM2*, *CYCS*, *OXA1L*, *PC*, and *LDHB* (Figure 10B). The metabolite–metabolite network contains 418 nodes and 907 edges with degree values exceeding 10, including pyruvic acid, citric acid, succinic acid, L-glutamine, L-aspartic acid, L-arginine, arachidonic acid, PGE2, isocitric acid, phytanic acid, hexacosanoic acid, adenosine triphosphate, and coenzyme A (Figure 10C).

## 4 Discussion

The adjuvant-induced arthritis (AA) model is one of the classical models for elucidating the pathogenesis of RA and assessing drug efficacy. These adjuvants are normally categorized into CFA and incomplete Freund’s adjuvant (IFA), where the main difference is that CFA contains heat-killed *Mycobacterium tuberculosis*. When using CFA to establish the model, the *M. tuberculosis* that contains molecular structures similar to those of synovial glycoprotein molecules elicits a cross-immune response and then activates excessive proliferation of T lymphocytes, which subsequently attack the C-antigen components in articular cartilage (Bordy et al., 2021). This process produces large quantities of inflammatory cytokines, such as TNF-α and IL-1β, leading to persistent inflammation. Notably, CFA containing heat-killed *M. tuberculosis* elicits robust Th1-biased cellular immune responses. This method offers advantages like simple operation and fast mold preparation. During the onset of diseases in animals, inflammation is first noted at the injection site, followed by joint swelling, swollen toes, and gradual worsening of the conditions, leading to impaired mobility in other parts of the body. The pathological manifestations include synovial proliferation, lymphocyte infiltration, and cartilage degeneration, which are similar to those observed in human RA (Lu et al., 2022; Shen et al., 2022). In the present study, the disordered metabolic pathways in the RA animal model were mainly divided into six categories as follows: amino acid metabolism pathways, lipid metabolism pathways, energy metabolism pathway, glycolysis pathway, pyrimidine metabolism pathway, and others (steroid hormone biosynthesis and primary bile acid biosynthesis).

### 4.1 Amino acid metabolism pathways

Previous research has shown that inducible nitric oxide synthase (iNOS) and NO are highly expressed in the serum, cartilage tissue, and synovial cells of RA patients. Arginine metabolism in the body is mainly catalyzed by NOS to produce NO, which then stimulates the growth of T lymphocytes and release of cytokines to promote inflammation (Shi et al., 2019). The concentration of glutamate in the synovial fluid in RA is closely related to the degree of joint swelling, bone erosion, and pain sensitivity; it can stimulate the proliferation of synovial fibroblasts, damage joint cartilage, and cause macrophages to release various cytokines to promote inflammatory responses (Hinoi and Yoneda, 2011; Lunec and Blake, 1985). At the same time, glutamate participates in the transport of dehydroascorbic acid, and its concentration is significantly increased in the serum of RA patients; this reflects the increased antioxidant and free-radical scavenging activities of vitamins in RA and is related to the degree of joint inflammation (McNulty et al., 2005; Firestein and McInnes, 2017). After treatment with ICA, the glutamate content was found to have decreased in RA animals, indicating that ICA regulates the glutamate metabolism pathway, reduces the expression of inflammatory factors, and exerts anti-inflammatory and analgesic effects. In RA, the deficiency of mitochondrial aspartic acid in the T cells can lead to increase in the size of endothelial cells and excessive production of the inflammatory factor TNF, thereby causing tissue inflammation (Narasimhan et al., 2018; Wu et al., 2021). Dysfunctional glutamine metabolism is known to promote the development of degenerative bone diseases, such as osteoporosis and osteoarthritis. Another report suggests that inhibiting the long non-coding RNA *NEAT1* can regulate glutamine metabolism mediated by *miRNA-338-3p*, leading to dysfunction of fibroblast-like synovial cells in RA (Zhou et al., 2019; Zhang et al., 2022).

### 4.2 Lipid metabolism pathways

Arachidonic acid is an important fatty acid in the human body that can be catalyzed by cyclooxygenase (COX) and lipoxygenase (LOX) to produce various inflammatory factors, such as PGE2, leukotriene B4 (LTB4), and thromboxane. It mediates the inflammatory responses of RA, increases vascular elasticity, and regulates blood cell functions. Studies have shown that calcium signaling regulated by arachidonic acid in the T cells of RA patients promotes synovial inflammation (Yang et al., 2018b; Ye et al., 2021). PGE2 can increase the production of IL-17A and expressions of CD80 and CD86 on gamma delta T cells in RA patients (Du et al., 2020). In the present study, the levels of arachidonic acid and PEG2 produced through metabolism were significantly higher in the serum of RA rats. After intervention with ICA, the levels of arachidonic acid and PEG2 were significantly decreased, indicating that ICA regulates the arachidonic acid metabolism pathway, reduces the expressions of inflammatory factors, and exerts anti-inflammatory and analgesic effects.

Lysophospholipids, including lysophosphatidylcholine (LPC) and lysophosphatidylethanolamine (LPE), are a rich species of lipids that primarily act as transporters of free fatty acids (Fu et al., 2016). LPC is one of the main products of the hydrolysis of glycerophospholipids by phosphatase A2 and plays important roles in cell proliferation and inflammatory responses. The binding of LPC to G-protein-coupled receptors can induce migration of T lymphocytes and macrophages, promote production of inflammatory cytokines, induce oxidative stress, and promote cell apoptosis, thus aggregating inflammation. Research has shown that lipid mediators play crucial roles in the pathogenesis of RA, where LPC is mainly involved in regulating immunity, inducing production of inflammatory factors, and promoting inflammatory responses (Liu et al., 2020; Koh et al., 2022). As the structural core of sphingophospholipids, Cer is believed to have crucial roles in the development of inflammation. Recent evidence shows that Cer accumulation enhances COX-2 expression and PEG2 liberation, resulting in various inflammatory diseases (Luan et al., 2023; Zhang et al., 2017; Claycombe et al., 2002). In the present study, we found that the levels of LysoPC(15:0), LysoPC(18:0), LysoPC(18:1), LysoPE(0:0/20:0), and Cer(d18:0/18:0) decreased in RA rats, indicating lipid metabolism disorder and significant inflammatory responses in the body.

### 4.3 Energy metabolism pathway

The citrate cycle occurring in the mitochondria is a critical link in the metabolism of carbohydrates, lipids, and amino acids. The intermediates of the citrate cycle are also precursors of many biosynthetic pathways, and the rate of the citrate cycle reflects the state of energy metabolism. It has been reported that RA-induced systemic inflammation can lead to increased energy metabolism (Zhou et al., 2016; Deng et al., 2016). Succinic acid as an intermediate product of the tricarboxylic acid cycle has been shown to increase the level of DNA and histone demethylase, leading to changes in the gene stability of tumor tissues and promotion of tumor occurrence and development. At the same time, the tumor necrosis factor-related protein 1in tumor tissues can promote tumor growth by inhibiting the activity of succinate dehydrogenase (Schaefer et al., 2018). In this work, the levels of citric acid and isocitric acid in the RA model group were decreased compared to those of the control group, indicating insufficient energy supply. This is consistent with the inflammatory responses inducing increased energy generation, mobilization, and consumption, leading to decreased energy metabolism in the body. The bodily levels of succinic acid increase in RA, indicating that the body is in an inflammatory environment.

### 4.4 Glycolysis pathway

Glycolysis metabolism is another metabolic pathway related to energy metabolism and plays a critical role in supplying ATPs. Evidence indicates that the synovial tissues in RA have increased glycolytic activities, leading to an acidic microenvironment that further induces the transformation of normal synovial cells. Enhanced glycolysis is related to hypoxia in the RA synovial membranes. Pyruvate is a substrate of the RA synovium metabolism and stimulates abnormal cell proliferation, angiogenesis, and pannus formation. In this study, we found that the pyruvate levels significantly decreased during disease progression, indicating weakened glycolytic activity (Chang and Wei, 2011; Zuo et al., 2021; Budzko et al., 2017).

### 4.5 Others

Neuroendocrine changes are some of the key factors resulting in RA progression and development. Some earlier studies reported that estradiol has protective anti-inflammatory abilities against arthritis in ovarian failure, especially in menopausal women, which may be mediated through the G-protein-coupled receptor. Estradiol was found to mitigate rat articular chondrocyte damage by targeting *ASIC1A*-mediated apoptosis and pulsed bone marrow mesenchymal stem cells in AA. In this study, the levels of 17a-estradiol produced by metabolism were significantly lower in the serum of RA rats (Schneider et al., 2019; Ding et al., 2009; Santen and Simpson, 2019; Song et al., 2020; Jahantigh et al., 2023). Bile acids are an important form of cholesterol clearance in the body (Shiomi, 2018) that play crucial roles in maintaining cholesterol homeostasis, preventing triglyceride accumulation, and promoting intestinal absorption of lipids and fat-soluble nutrients. Owing to the decrease in bile acid secretion, the inhibition of vitamin D hydration and absorption can lead to secondary osteoporosis. In this study, the levels of taurochenodeoxycholate, taurocholic acid, and glycochenodeoxycholate were significantly lower in the serum of RA rats, indicating that the bile acid metabolism was altered in RA after ICA treatment. Vitamin K2 is an essential substance available in small quantities from organic compounds that induces not only bone mineralization of human osteoblasts and apoptosis of osteoclasts but also apoptosis of RA synovial cells and mitogen-activated peripheral blood mononuclear cells (Ebina et al., 2013; Xu et al., 2021). Phytanic acid is a trimethyl-saturated branched-chain fatty acid known to prevent metabolic disorders that may have the ability to stimulate skeletal muscle capacity consumption (Che et al., 2013). If the alpha oxidase system in the body is defective, then phytanic acid cannot be degraded and will be accumulated in the blood and brain. In this study, phytanic acid levels were reduced in the RA model, indicating that RA cells may enhance the alpha oxidase system, resulting in increased consumption of phytanic acid and metabolic disorders in the body thereof.

## 5 Conclusion

In the present study, we adopted a non-targeted metabolomics strategy for analyzing serum samples obtained from SD rats with RA. The anti-RA mechanisms of ICA treatment may be mediated by ameliorating the levels of 23 metabolites, including 17a-estradiol, 5-methylcytidine, arachidonic acid, arginine, aspartic acid, cer(d18:0/18:0), citric acid, glutamine, and glycochenodeoxycholate; these metabolites are involved in 10 biological pathways related to alanine, aspartate and glutamate metabolism; arachidonic acid metabolism; citrate cycle; pyruvate metabolism; and glycolysis/gluconeogenesis pathways that are known to regulate the oxidative stress state and produce inflammatory effects.

## Data Availability

The original contributions presented in this study are included in the article/Supplementary Material, and any further inquiries may be directed to the corresponding authors.
