# Supplementary material for: High-throughput metabolomics exploring the pharmacological effects and mechanism of icariin on rheumatoid arthritis rat based on ultrahigh-performance liquid chromatography coupled with quadrupole time-of-flight mass spectrometry
Source: Front Mol Biosci. 2025 Apr 9;12:1514882. doi: 10.3389/fmolb.2025.1514882 (PMC12015166; doi:10.3389/fmolb.2025.1514882)
Supplement: Supplementary file 1 [file Table1.docx]

**Table S1** Differential metabolites among control, RA model, methotrexate-treated and ICA-treated group

| **No.** | **Rt(min)** | **Ion Mode** | **Formula** | **m/z** | **Metabolites Name** | **HMBD** | **M/C Trend** | **Regulated by ICA** |
| --- | --- | --- | --- | --- | --- | --- | --- | --- |
| 1 | 0.38 | M-H | C3H6O3 | 89.0238 | Lactic acid | HMDB01311 | ↑ |  |
| 2 | 0.54 | M+H | C18H24O2 | 545.3620 | 17a-Estradiol | HMDB0000429 | ↓ | **√** |
| 3 | 0.77 | M+H | C10H15N3O5 | 280.0910 | 5-Methylcytidine | HMDB0000982 | ↓ | **√** |
| 4 | 1.05 | M-H | C20H32O2 | 303.2296 | Arachidonic acid | HMDB01043 | ↑ | **√** |
| 5 | 1.33 | M+H | C6H14N4O2 | 175.1193 | Arginine | HMDB00517 | ↓ | **√** |
| 6 | 1.56 | M-H | C4H7NO4 | 132.0305 | Aspartic acid | HMDB0000191 | ↓ | **√** |
| 7 | 2.28 | M-H | C36H73NO3 | 566.5473 | Cer(d18:0/18:0) | HMDB11761 | ↓ | **√** |
| 8 | 2.37 | M+H | C6H8O7 | 215.0151 | Citric acid | HMDB00094 | ↓ | **√** |
| 9 | 2.45 | M-H | C21H30O4 | 345.2052 | Corticosterone | HMDB01547 | ↓ |  |
| 10 | 3.43 | M-H | C5H10N2O3 | 145.0613 | Glutamine | HMDB00641 | ↑ | **√** |
| 11 | 3.81 | M+H | C8H20NO6P | 258.1111 | Glycerophosphocholine | HMDB00086 | ↓ |  |
| 12 | 3.92 | M-H | C32H51NO11 | 448.3075 | Glycochenodeoxycholate | HMDB0000637 | ↑ | **√** |
| 13 | 4.06 | M+H | C6H8O7 | 215.0100 | Isocitric acid | HMDB00193 | ↓ | **√** |
| 14 | 4.42 | M+H | C4H8NO7P | 214.0060 | L-Aspartyl-4-phosphate | HMDB0012250 | ↑ | **√** |
| 15 | 4.77 | M-H | C5H9NO4 | 146.0460 | L-Glutamate | HMDB0000641 | ↓ | **√** |
| 16 | 4.93 | M+H | C6H14N2O2 | 147.1100 | Lysine | HMDB00182 | ↑ |  |
| 17 | 5.21 | M-H | C23H48NO7P | 480.3138 | LysoPC(15:0) | HMDB10381 | ↓ | **√** |
| 18 | 5.33 | M-H | C26H54NO7P | 522.3559 | LysoPC(18:0) | HMDB10384 | ↓ | **√** |
| 19 | 6.08 | M-H | C4H6O4 | 117.0194 | Succinic acid | HMDB0000254 | ↑ | **√** |
| 20 | 6.24 | M-H | C26H52NO7P | 520.3399 | LysoPC(18:1) | HMDB02815 | ↓ | **√** |
| 21 | 6.57 | M+H | C40H78NO8P | 732.5558 | PE(15:0/20:1) | HMDB08900 | ↓ |  |
| 22 | 6.63 | M+H | C20H40O2 | 330.3360 | Phytanic acid | HMDB0000801 | ↓ | **√** |
| 23 | 7.08 | M+H | C20H32O5 | 353.2353 | Prostaglandin E2 | HMDB01220 | ↑ | **√** |
| 24 | 7.21 | M-H | C3H4O3 | 87.0079 | Pyruvic acid | HMDB00243 | ↓ | **√** |
| 25 | 7.50 | M+H | C18H38NO5P | 380.2578 | Sphingosine | HMDB0000277 | ↓ |  |
| 26 | 7.92 | M+H | C25H52NO7P | 510.3602 | LysoPE(0:0/20:0) | HMDB11481 | ↓ | **√** |
| 27 | 8.26 | M-H | C26H45NO6S | 498.2889 | Taurochenodeoxycholate | HMDB0000951 | ↑ | **√** |
| 28 | 8.58 | M-H | C26H45NO7S | 514.2828 | Taurocholic acid | HMDB00036 | ↑ | **√** |
| 29 | 8.77 | M-H | C9H11NO3 | 180.0666 | Tyrosine | HMDB0000158 | ↑ |  |
| 30 | 9.24 | M-H | C9H12N2O6 | 243.0615 | Uridine | HMDB00296 | ↓ |  |
| 31 | 9.43 | M+H | C21H26O2 | 311.4299 | Vitamin K2 | HMDB0060487 | ↓ | **√** |

Note: “↑” represents an upward trend, and “↓” represents a downward trend.
